# When the Idiom Advantage Comes Up Short: Eye-Tracking Canonical and Modified Idioms

**DOI:** 10.3389/fpsyg.2021.675046

**Published:** 2021-08-02

**Authors:** Marianna Kyriacou, Kathy Conklin, Dominic Thompson

**Affiliations:** School of English, University of Nottingham, Nottingham, United Kingdom

**Keywords:** idioms, idiom modification, adjective insertion, reading, eye-tracking, processing advantage, context, meaning integration

## Abstract

The literature on idioms often talks about an “idiom advantage,” such that familiar idioms (*spill the beans*) are generally processed faster than comparable literal phrases (*burn the beans*). More recently, researchers have explored the processing of idiom modification and while a few studies indicate that familiarity benefits the processing of modified forms, the extent of this facilitation is unknown. In an eye-tracking study, we explored whether familiar idioms and modified versions with 1 or 2 adjectives {*spill the [spicy, (red)] beans*} are processed faster than matched literal phrases {*burn the [spicy, (red)] beans*} when both were preceded by a biasing context. The results showed that adjectives inserted in idioms induced longer fixations and were more likely to elicit a regression. However, idiom verbs and final words were processed with the same ease in all adjective conditions, implying that modifying idioms did not impede their processing. In contrast to the widely reported “idiom advantage,” the results demonstrated that canonical and modified idioms were *slower* to read relative to matched literal controls. This was taken to reflect the competition between an idiom’s literal and figurative meaning, and subsequently the need to select and integrate the contextually appropriate one. In contrast, meaning integration in literal, unambiguous phrases was easier. We argue that processing costs associated with meaning selection may only manifest when idioms are preceded by a biasing context that allows disambiguation to occur in the idiom region, and/or when literal control phrases are contextually appropriate and carefully matched to idioms. Thus, idiom recognition/activation may elicit the well attested idiom advantage, while meaning selection and integration may come at a cost, and idiom modifications may simply add to the cognitive load.

## Introduction

Idioms such as *weather the storm* (“overcome a difficulty”) have a canonical form (Wray, 2002, 2012; Van Lancker Sidtis, 2004, 2012) in which they often exhibit a processing advantage over fully compositional, literal phrases (*avoid the storm*) (Siyanova-Chanturia and Martinez, 2015). Despite their conventionality, idioms are modifiable (e.g., Moon, 1998; Bybee, 2000; Schmitt, 2005; Langlotz, 2006; Duffley, 2013; Hovhannisyan and Mkrtchyan, 2014). For example, adjectives can be inserted, modifying the figurative meaning, as in *weather a renewed storm* (Moon, 1998). Here, the inserted adjective *renewed* suggests that the difficulty had occurred and been dealt with before, or equally, that a new difficulty arose following a previous one. Modified idioms have recently attracted interest in psycholinguistics (Omazic, 2008; Dronov and Bochaver, 2014; Haeuser et al., 2020; Kyriacou et al., 2020; Mancuso et al., 2020), but we still know very little about the way they are processed. The present study aims to address this by comparing the processing of canonical idioms and idioms modified by adjectives, exploring whether modified idioms benefit from a processing advantage relative to matched literal phrases.

A considerable literature reports a processing advantage for canonical idioms (*spill the beans*) over literal phrases (*spill the chips*), regardless of whether they are intended figuratively or literally (Siyanova-Chanturia et al., 2011; Carrol, 2021). This has been demonstrated by faster response times in lexical decision tasks (Ortony et al., 1978; Swinney and Cutler, 1979; Tabossi et al., 2009; Rommers et al., 2013; Carrol and Conklin, 2014b), shorter reading times in self-paced reading tasks (Gibbs, 1980; Conklin and Schmitt, 2008; Colombo, 2014), shorter reading times, fewer regressions, and increased skipping rates (for idiom final words) in eye-tracking studies (Underwood et al., 2004; Siyanova-Chanturia et al., 2011; Carrol and Conklin, 2017, 2020), and shorter durations in production (Van Lancker et al., 1981). ERP studies have reported reduced N400 and increased P300 amplitudes for idioms (Strandburg et al., 1993; Laurent et al., 2006; Vespignani et al., 2010; Rommers et al., 2013), reflecting stimulus predictability (e.g., *horns* in *take the bull by the*) (Kutas and Hillyard, 1980, 1984; Van Petten and Kutas, 1990; Hagoort et al., 2004), and expectancy confirmation, respectively (i.e., matching what is being encountered to what is already known and stored as a template in memory) (Verleger, 1988).

Although the idiom processing advantage was originally taken to indicate that idioms were stored holistically in the mental lexicon and were retrieved without undergoing compositional analysis (Bobrow and Bell, 1973; Swinney and Cutler, 1979; Wray, 1992; Wray, 2002; Ellis, 1996), later findings demonstrated that syntactic analysis occurs during idiom processing (e.g., Cacciari and Tabossi, 1988; Cutting and Bock, 1997; Peterson et al., 2001; Snider and Arnon, 2012; Holsinger, 2013; Holsinger and Kaiser, 2013; Cacciari and Corradini, 2015). As a result, idioms are thought to have a hybrid representation, operating partly as lexical items and partly as compositional phrases (Cacciari and Tabossi, 1988; Sprenger et al., 2006; Libben and Titone, 2008). An idiom processing advantage arises when an idiomatic phrase is recognized and activated early (i.e., before the phrase offset), at which point compositional analyses may stop in favor of the (faster) lexical retrieval route. In contrast, matched literal phrases are slower to process because they require a full compositional analysis until the phrase offset.

Models offer explanations for why idiom recognition and activation occur at different points. The Configuration Hypothesis (Cacciari and Tabossi, 1988) postulates that recognition is determined by predictability. For example, after encountering the fragment *hit the nail*, one might recognize the predictable idiom *hit the nail on the head*, leading to fast retrieval of the figurative meaning, without the need to process any of the constituents after the recognition point (the idiom key) (Cacciari and Tabossi, 1988; Cacciari et al., 2007; Tabossi et al., 2009; Fanari et al., 2010; Cacciari and Corradini, 2015). Conversely, unpredictable idioms are recognized late, and their figurative meaning becomes available *after* the phrase offset (Cacciari and Tabossi, 1988; Fanari et al., 2010). In this case, matched literal phrases might demonstrate a processing advantage since their meaning would be available *at* phrase offset, not after it; however, no studies to date have directly explored this question. The Multidetermined Model (Libben and Titone, 2008) maintains that idiom recognition depends on additional factors, including literalness (i.e., how plausible the literal meaning of an idiom is), transparency/decomposability (i.e., how guessable/analyzable the figurative meaning is), as well as familiarity and frequency. It further assumes that these factors may interact or weigh in at different points during processing. For example, greater frequency and familiarity may lead to early idiom recognition, while literal plausibility may delay recognition due to the (equal) viability of the literal meaning of the phrase (Titone and Libben, 2014; Mancuso et al., 2020), and transparency may affect idiom processing at later stages (i.e., after the phrase offset). However, studies have shown that high literalness facilitates rather than hinders idiom processing (Mueller and Gibbs, 1987; Cronk and Schweigert, 1992; Beck and Weber, 2020), but an effect of transparency is less conclusive. Some studies report a processing benefit for transparent idioms (Gibbs and Nayak, 1989; Gibbs et al., 1989; Caillies and Butcher, 2007), others for less transparent ones (Titone and Libben, 2014; Carrol and Conklin, 2020), and yet some others report a null effect (Van de Voort and Vonk, 1995; Libben and Titone, 2008; Tabossi et al., 2008; Carrol and Conklin, 2014b).

Familiarity is the most consistently facilitative factor in idiom processing and perhaps the major contributor to the idiom processing advantage (Schweigert, 1986; Cronk and Schweigert, 1992; Tabossi et al., 2009; Van Lancker Sidtis, 2012; Carrol and Littlemore, 2020). Accessing the figurative meaning of an idiom is conditional upon the phrase being “known” (i.e., being familiar), as a pure compositional analysis will result in a literal understanding [*spill* + *the* + *beans*≠ “reveal the secret(s)”]. It is thought that familiarity may in fact be so powerful, as to mask potential effects from other factors (Abel, 2003; Carrol et al., 2018). Libben and Titone (2008), for example, found that transparency only influenced meaningfulness judgments if idioms were less familiar. Crucially, as idioms are recurring phrases with a conventionalized form and meaning, they tend to become highly familiar to native speakers (Siyanova-Chanturia and Martinez, 2015) despite being relatively infrequent in language (Moon, 1998). Thus, idioms are often recognized and processed faster than novel literal phrases, simply because they are fundamentally (more) familiar expressions (Carrol and Conklin, 2020).

While the idiom processing advantage is a well-established phenomenon for (at least) familiar and canonical idioms, little is currently known about the processing of modified forms. Recent psycholinguistic studies have demonstrated that modified idioms preserve their figurative meaning (Kyriacou et al., 2020; Mancuso et al., 2020), despite earlier linguistic views to the contrary (e.g., Fraser, 1970; Nunberg, 1978; Gibbs and Nayak, 1989). However, idiom modification and its impact on the idiom processing advantage has not been explored systematically. Further, models do not make clear predictions about how modification impacts processing relative to unmodified forms or matched literal phrases. What is more, recent evidence suggests that modified forms benefit from the familiarity of the canonical form, despite their otherwise “novel” presentation. For example, although passive idiom forms are extremely rare in language use, passive forms of more familiar (and frequent) idioms were faster to read than those of less familiar (and less frequent) idioms (Kyriacou et al., 2020). This suggests that modifications do not necessarily render an idiom unfamiliar; it may be the co-occurrence of an idiom’s components (and not necessarily their precise order) that really establishes a phrase as a familiar expression. Further, assuming that familiarity is the main driver of the idiom advantage, it may be that modified versions of familiar idioms will exhibit an advantage over matched literal phrases^1^.

In this study we focus on word, and more specifically adjective, insertion. Crucially, adjective insertion appears to be more acceptable than other types of idiom modification (Geeraert et al., 2017a). Further, findings from other types of formulaic sequences have demonstrated that a formulaic advantage survives a similar manipulation. For example, Molinaro et al. (2013) found that the nouns in complex prepositions following adjectival insertion were read more slowly but induced significantly smaller N400 amplitudes than the same nouns in canonical forms (*in the capable hands of* versus *in the hands of*). The researchers concluded that the inserted adjectives restricted the range of possible phrase continuations, which increased the cloze probability of the nouns, yielding easier integration in the modified condition. In an eye-tracking study, Vilkaitė (2016) found that collocations, with and without inserted words [*provide (some of the) information*], were both faster to read than matched non-collocations [*compare (some of the) information*]. The findings demonstrate that a formulaic advantage extends to modified forms over matched non-formulaic sequences.

Some early behavioral studies looking at word insertion in idioms suggested that lexical insertion may neutralize the idiom advantage or even induce a processing cost relative to literal phrases. For example, idioms with added words (*he didn’t spill a single bean*) were read at the same rate as their literal paraphrases (*he didn’t say a single word*) in a self-paced reading task (McGlone et al., 1994). Further, final words in modified idioms (e.g., *bag* in *he let the fat cat out of the bag*) elicited longer reaction times than the same words in both canonical idioms (*he let the cat out of the bag*) and literal control phrases (*he let the fat cat out of the closet*) (Van de Voort and Vonk, 1995). However, behavioral tasks requiring a decision and/or button press might not best represent processing in more natural contexts. Indeed, findings from an eye-tracking study by Geeraert et al. (2017b) show that idiom variants are not necessarily more difficult to process than canonical forms, although they require more time to read the additional words (*hear something through the judgmental grapevine*). However, in their study, matched literal control phrases were not included.

In a recent eye-tracking study, Haeuser et al. (2020) explored the processing of idioms with and without inserted adjectives (by younger and older adults). Idioms were followed by context which biased toward the figurative or literal understanding of the phrase [e.g., *Larry bit the (iron) bullet* (*and bought diamond earrings for his wife’s birthday*)/(*to verify the quality of casings on his ammunition*)]. Literal control phrases were constructed by replacing the idiom verb and were equally plausible in their context [*Larry hid the (iron) bullet so the police would not find the crucial evidence*]. The researchers observed early facilitation for the final word of canonical idioms, as fixations were shorter during first pass gaze duration (thus alluding to an idiom processing advantage). However, in total reading time, canonical idioms were only faster to read if they were highly familiar; low-familiar idioms were read more slowly than matched literal phrases. Importantly, modified idioms were read significantly more slowly than matched literal phrases only when used in their figurative sense. Modified idioms in their literal sense did not differ from matched literal phrases and were significantly faster to read than the (same) modified idioms in their figurative sense. Based on this, the researchers argued that modified idioms were first computed literally and, as a result, processing difficulties arose when subsequent context invalidated this interpretation. However, a stronger advantage for canonical forms than the one reported in Haeuser et al. (2020) and potentially an advantage for adjectivally modified idioms may arise if *prior* context biases the figurative meaning of the phrases, boosting not only the recognition and activation of the idioms, but also reducing (or eliminating) the concurrent activation of the literal meaning.

From the discussion thus far, it appears that the processing advantage in canonical idioms is (mostly) attributable to their familiarity. Evidence further suggests that modified versions benefit from the familiarity of the canonical form, implying that modified idioms are not necessarily perceived as unfamiliar expressions. In addition, word insertion is a more acceptable type of modification in idioms (than passivization, for instance), and one that has been found to come with a processing benefit in other types of formulaic sequences, with Vilkaitė (2016) showing that a formulaic advantage survives the addition of up to three intervening words. Therefore, it is hypothesized that familiar idioms modified with inserted adjectives may demonstrate a processing advantage relative to matched literal phrases when the prior context biases toward their figurative interpretation. To explore this, the current study included a manipulation where zero, one, or two adjectives were inserted (*spill the beans*; *spill the spicy beans*; *spill the spicy, red beans*). Matched literal phrases were created by changing the idiom verb {*burn the [spicy, (red)] beans*} and these were embedded in a context that biased their respective meaning.

Notably, adjectives in idioms have an unavoidable metaphoric interpretation and require inferencing. For instance, in a context about an illegitimate love affair, the addition of *spicy* and *red* in *spill the spicy, (red) beans* could be interpreted as an attempt to highlight the scandalous nature of the secrets. In contrast, both *spicy* and *red* in the literal phrase *burn the spicy, (red) beans* would simply refer to the literal properties of the beans. Thus, the adjectives in idioms may be semantically more complex, leading to relatively longer processing times. However, the current manipulation is not unlike that of Molinaro et al. (2013). Consider, for example, their item *in the capable hands of*. This complex prepositional phrase is non-literal, since being cared for does not entail being physically in one’s hands. Consequently, the modifier *capable* refers to the abilities of the agent – and not to skills involving dexterity. Molinaro et al. (2013) found that nouns following adjectival insertion elicited a reduced N400 relative to the same noun in canonical forms, presumably because the modifiers increased their cloze probability. A similar facilitation may be observed for idiom final words appearing after the manipulation in the 1- and 2-adjective conditions [e.g., *beans* in *spill the spicy, (red) beans*], relative to the same words in the unmodified condition (*beans* in *spill the beans*), but also relative to the same words in literal phrases [*beans* in *burn the spicy, (red) beans*], as idiom final words are likely to have a higher cloze probability than final words in literal phrases. If the addition of the adjectives further increases their cloze probability, this should further facilitate their processing relative to those in literal phrases. However, this potential facilitation might be offset by the cost of the metaphorical interpretation of the adjectives. That is, shorter fixations for idiom final words might not result in faster reading times for idioms as a whole. For this reason, it is important to look for an idiom advantage not only for the phrase as a region, but also in the verb, adjective, and final word region separately.

We hypothesized that when idioms are familiar and preceded by a context biasing their figurative meaning: (a) canonical forms should be faster to process than matched literal phrases, in accordance with the idiom processing advantage (Siyanova-Chanturia and Martinez, 2015), and (b) modified idioms with 1 or 2 adjectives are likely to be faster than matched literal phrases, although an advantage in this case might only manifest in the final word region. Moreover, we hypothesized that: (c) the final words of modified idioms should exhibit an advantage relative to the same words in canonical forms if the inserted modifiers significantly increase their cloze probability, and (d) that factors known to influence idiom processing (i.e., familiarity, frequency, predictability, transparency, and literalness) should modulate the processing of modified forms, in line with previous research (Kyriacou et al., 2020).

## Materials and Methods

### Participants

Ninety native speakers of British English (mean age = 22 years, SD = 5.5; 18 males, 72 females) took part in the eye-tracking experiment. Participants received course credit or compensation for their participation. This study was reviewed and approved by the Faculty of Arts Ethics of the University of Nottingham, and participants signed a consent form prior to participating.

### Materials

Ninety idioms, comprised of a verb, determiner/pronoun, and noun (*spill the beans/find your feet*) were selected from the Collins COBUILT Idioms Dictionary (Sinclair, 2011). Idioms were matched with a literal control phrase by substituting the verb of the idiom (*burn the beans/rub your feet*). Verbs were matched for frequency [*t*(89) = 0.91, *p* = 0.36]^2^, but idiom verbs were significantly shorter [*t*(89) = −2.94, *p* = 0.004]. However, both variables were considered in relevant models to account for any differences. The characteristics of the items are presented in Table 1 and the full list of items can be found in the Supplementary Appendix.

**TABLE 1:** Summary of item characteristics.

	Phrase type	
	Idioms	Literal phrases
	Mean (SD)	Mean (SD)
**Verbs**		
Length	5.52 (1.67)	6.22 (1.79)
Frequency	4.38 (0.79)	4.28 (0.73)
**Phrases**		
Familiarity	4.26 (0.81)	–
Literalness	5.56 (0.84)	–
Transparency	2.89 (0.68)	–
Frequency	0.32 (0.31)	0.15 (0.51)
Predictability	0.43 (0.35)	0.32 (0.33)

*Length is reported in number of characters, frequency on the Zipf scale, familiarity and transparency assessed in norming tasks, on a scale from 1 (less familiar/more transparent) to 5 (more familiar/less transparent), and literalness from 1 (most likely literally) to 7 (least likely literally). Cloze probability, assessed in cloze completion tasks, is reported as probability between 0 and 1. Familiarity, transparency, and literalness scores are only reported for idioms as they do not apply to the literal phrases.*

For both Phrase Types (idioms and literal phrases), we calculated frequency and predictability (used interchangeably with cloze probability). Both idioms and literal phrases were of relatively low frequency, but idioms were significantly more frequent than literal phrases [*t*(89) = 4.41, *p* < 0.001]. Predictability was measured as the cloze probability of the final words of the phrases in context, via a completion task with 30 participants. The items were divided in two lists to counterbalance across Phrase Type (literal versus figurative phrase) and included the stimuli sentences leading up to (but excluding) the final words of the phrases of interest. Thirty participants from a similar population to that of the main study were asked to complete the phrases with the first word that came to mind. Paired *t*-tests revealed that final words in idioms had a significantly higher cloze probability than (the same words) in the literal phrases [*t*(89) = −2.16, *p* = 0.03], although cloze probability was fairly low in both Phrase Types (43 and 32%, respectively).

For idioms only, we collected norming data on familiarity (*n* = 16), transparency (*n* = 16), and literalness (*n* = 16) from another 48 participants who did not take part in the main study but were from a similar population. Familiarity was defined as familiarity with the figurative meaning of an idiom (a dictionary definition was provided for this) and was judged on a scale from 1 (very unfamiliar with the meaning) to 5 (very familiar with the meaning). Transparency was defined as how guessable the figurative meaning was, based on the idiom component words alone, and was judged on a scale from 1 (very easy to guess) to 5 (very hard to guess). Because research has shown that familiarity can influence judgments of transparency (Carrol et al., 2018), no idiom definitions were provided for this task. Literalness was defined as how likely it is to encounter each phrase literally versus figuratively on a scale from 1 (most likely literally) to 7 (most likely figuratively), as a means of gauging idioms’ literal plausibility as well as meaning dominance. As can be seen in Table 1, idioms were rated as mostly familiar and non-literal, as well as relatively transparent. Of note, all norming data were gathered for the idioms and literal phrases in their unmodified form (i.e., without adjectives) and were used in appropriate models as predictors for both unmodified and modified idioms and literal phrases, respectively.

The idiom-literal pairs (*n* = 180) were embedded in sentences where the context matched the figurative meaning of the idiom, or the literal meaning of the literal phrase, respectively. The phrases contained either 0, 1, or 2 extra adjectives, creating in total 540 items (270 idioms and 270 literal phrases). Phrase Type and Adjective Condition led to a 2 × 3 study design, resulting in a total of six experimental conditions (Table 2): (1) idiom without adjectives (ID0), (2) idiom with 1 adjective (ID1), (3) idiom with 2 adjectives (ID2), (4) literal phrase without adjectives (LIT0), (5) literal phrase with 1 adjective (LIT1), and (6) literal phrase with 2 adjectives (LIT2). The two words immediately preceding and following the phrases of interest (*he eventually* and *when he* in the example in Table 2) were kept constant across conditions to control for spillover effects, except in one instance (*pick/examine one’s brain*), where only one preceding word (as well as the two following ones) were the same across conditions.

**TABLE 2:** Example sentences for the idiom *spill the beans* and its literal control phrase *burn the beans*.

Condition	Example sentence
ID0	Oscar had always been terrible at keeping secrets, so *he eventually* **spilt the beans** *when he* was asked about his friend’s ongoing affair
ID1	Oscar had always been terrible at keeping secrets, so *he eventually* **spilt the spicy beans** *when he* was asked about his friend’s ongoing affair
ID2	Oscar had always been terrible at keeping secrets, so *he eventually* **spilt the spicy, red beans** *when he* was asked about his friend’s ongoing affair
LIT0	Oscar wanted to cook a homemade Mexican dish, but *he eventually* **burnt the beans** *when he* forgot to turn off the hob, so he ended up ordering pizza
LIT1	Oscar wanted to cook a homemade Mexican dish, but *he eventually* **burnt the spicy beans** *when he* forgot to turn off the hob, so he ended up ordering pizza
LIT2	Oscar wanted to cook a homemade Mexican dish, but *he eventually* **burnt the spicy, red beans** *when he* forgot to turn off the hob, so he ended up ordering pizza

*The phrases of interest (in bold) are split across Adjective Condition with ID0 and LIT0 denoting an idiomatic and literal phrase without adjectives, ID1 and LIT1 denoting an idiomatic and literal phrase with 1 adjective, and ID2 and LIT2 denoting an idiomatic and literal phrase with 2 adjectives, respectively.*

We used the same adjectives in both Phrase Types to control for semantic complexity and other word properties, though as we mentioned in the Introduction, adjectives in idioms would require inferencing. As we wanted to keep the adjectives congruous in their respective contexts, we selected ones that would be plausible in both scenarios. For instance, in Table 2, *spicy* could be interpreted as *scandalous*, while the red color is often associated with passion and danger, thus alluding to the illegitimate love affair described in the sentence. In the literal context, both *spicy* and *red* are plausible, as red, spicy beans are often found in Mexican food. In some instances, the adjectives had to be more generic to fit both contexts (*big*, *small*, etc.). Due to the large number of stimuli sentences (540), collecting norming data to judge the felicity of the adjectives in their respective phrases was prohibitive. However, the aptness of the adjectives in the literal versus the idiomatic expressions should be reflected in the reading times of the adjective region (and potentially the phrase region). Specifically, significantly longer fixations on idiom adjectives relative to the same adjectives in literal phrases would suggest insufficient aptness and/or difficult integration. Therefore, to test our hypotheses, and to account for differences in the processing of the adjectives, we set four regions of interest (ROIs): (1) phrase region, (2) verb region (*spill/burn*), (3) adjective region (*spicy/spicy, red*), and (4) final word region (*beans*). The verb region was included to check for any early idiom advantages at the phrase onset.

### Procedure

The stimuli sentences were divided across six lists using a Latin square design, such that each participant only saw an item in one of the six conditions. Care was taken so that ROIs (including the two preceding and following words) did not occur over a line break to avoid contamination from saccade programming (Conklin et al., 2018). Each list contained 90 experimental (45 idioms and 45 literal phrases) and 90 filler sentences that were of a similar structure. The fillers were either literal sentences or they contained other types of formulaic sequences (e.g., binomials, proverbs), some with inserted adjectives, so that the experimental items would not stand out.

An EyeLink 1000+ desktop-mount eye-tracker with a minimum sampling rate of 500 Hz was used. Participants were seated in front of a computer monitor with a chin- and forehead-rest to minimize head movement. The eye-tracker was calibrated using a 9-point grid, and re-calibration was performed, as necessary. The sentences were triple-line spaced, in black font (Courier New, size 14) on a white background, and were displayed in the middle of the screen, one at a time. Drift correction was performed before the presentation of each trial. Participants were asked to read the sentences as quickly as possible but for comprehension and to press ENTER to proceed from one trial to the next. Forty percent of the filler items were followed by a Yes/No comprehension question to ensure participants’ attention. The presentation of the sentences was randomized by participant.

## Results

The overall accuracy on the comprehension task was high (92%), indicating that the participants were engaged with the stimuli. Fixations shorter than 80 ms were incorporated to the largest nearest fixation (for a distance up to 0.5 degrees of visual angle) or were removed (0.06% of the data). A further 0.10% of the data was lost due to track loss and 0.39% due to the removal of outliers. Finally, some observations were lost due to skipping (verbs: 16.5%; adjectives: 18.82%; final words: 25%)^3^.

To make our results comparable to those of Haeuser et al. (2020), and in line with Carrol and Conklin’s (2014a) suggestions, we examined both early and late eye-tracking measures for both the whole phrase and individual words. For the phrase, we examined two late measures: total reading time (duration of all fixations and refixations on ROI) and regression probability (the probability for a regression into the ROI from later parts of the sentence). For individual words (verb, adjective, and final word), we examined two early measures: first pass gaze duration (duration of all fixations and refixations in the ROI from when the ROI is first fixated during first pass reading, and until the eye moves to the right) and go-past time (duration of all fixations and refixations in the ROI from when the ROI is first fixated and before the eye moves to the right, including refixations coming from the left of the ROI and any time spent in previous parts of the sentence), and one late measure: total reading time^4^.

The data were analyzed using mixed-effects models and the *lme4* package, version 1.1-21 (Bates et al., 2014) in R, version 4.0.3 (R Core Team, 2020). Reading times (first pass gaze duration, go-past time, and total reading time) were log-transformed and analyzed using linear mixed-effects models, while regression probability (a binary variable) was analyzed using logistic regression (Jaeger, 2008). Phrase Type and Adjective Condition were incorporated in the models as fixed effects: Phrase Type as a two-level factor (literal phrase and idiom) and Adjective Condition as a three-level factor (0-adjectives, 1-adjective, and 2-adjectives), except for the adjective region where Adjective Condition was set as a 2-level factor (1-adjective and 2-adjectives) since there were no adjectives in unmodified conditions. The literal phrase and 0-adjective condition were each set as the respective baselines in all analyses, except for the adjective region analyses, where the literal and 1-adjective condition were set as the baselines. Due to model convergence issues, the models for the Phrase Region, the Go-Past model for the Verb Region, and the First Pass Gaze Duration model for the Adjective Region included a by-item and by-subject random intercept and slope for the Phrase Type only, but not the Adjective Condition, while all remaining models only included by-subject and by-item intercepts without slopes (Barr et al., 2013; Bates et al., 2015). Additive models were initially fitted, and interactions were included only if they significantly improved the models’ fit. All model means (Table 3) and pairwise comparisons between Phrase Type and Adjective Condition, as well as pairwise comparisons between the three levels of the Adjective Condition, were calculated using the *emmeans* package (Lenth, 2018), and are reported using Bonferroni correction. All model outputs are provided as Supplementary Material to the manuscript.

**TABLE 3:** Model means collapsed across ROI and condition.

	Total reading time		Regression probability			
	Mean	SE	Mean	SE		
**Phrase region**						
LIT0	**546**	17.3	0.24	0.01		
ID0	**591**	21.1	0.28	0.02		
LIT1	**681**	19.6	**0.32**	0.02		
ID1	**738**	24.0	**0.40**	0.02		
LIT2	**781**	25.1	**0.36**	0.02		
ID2	**846**	29.7	**0.49**	0.02		

	**First pass gaze**		**Go-past time**		**Total reading time**	
	**Mean**	**SE**	**Mean**	**SE**	**Mean**	**SE**

**Verb region**						
LIT0	226	3.98	259	6.15	**253**	5.90
ID0	227	4.02	261	6.41	**266**	6.23
LIT1	227	3.99	260	6.17	**260**	6.08
ID1	228	4.04	261	6.43	**273**	6.42
LIT2	228	4.01	264	6.26	**266**	6.19
ID2	230	4.05	266	6.52	**279**	6.53
**Adjective region**						
LIT1	234	4.13	**257**	5.37	**259**	5.75
ID1	239	4.24	**273**	5.69	**273**	6.05
LIT2	232	3.92	**251**	4.93	**254**	5.41
ID2	238	4.02	**266**	5.22	**268**	5.69
**Final word region**						
LIT0	216	3.55	**232**	4.64	**230**	4.53
ID0	218	3.58	**245**	4.89	**237**	4.67
LIT1	218	3.61	**238**	4.77	**235**	4.66
ID1	221	3.64	**251**	5.02	**242**	4.79
LIT2	222	3.67	**242**	4.84	**237**	4.69
ID2	225	3.70	**255**	5.09	**245**	4.82

*Reading times (total reading time, first pass gaze, and go-past time) are reported in milliseconds. Regression probability is reported as probability between 0 and 1. Values in bold indicate significant pairwise comparisons (*p* ≤ 0.05) between ID0 and LIT0, ID1 and LIT1, or ID2 and LIT2, respectively.*

Familiarity, literalness, transparency, cloze probability, phrase/verb frequency, were added stepwise in relevant models as predictors and were only kept if they significantly improved the model’s fit. Checks indicated problematic multicollinearity (κ = 31) as idiom familiarity correlated with both transparency (*r* = −0.59) and cloze probability (*r* = 0.49). We centered the predictors, and further residualized familiarity against transparency and cloze probability (*r*s < ± 0.001). The κ value dropped to 1.6 suggesting no further collinearity and residualized familiarity still correlated highly with its original variable (*r* = 0.73). Aside from these factors, we also considered phrase/word length as appropriate for each ROI, and trial sequence number to ensure that the main effects were not driven by length, or by increased exposure and accumulated experience with the stimuli over the course of the experiment.

### Phrase-Level Analyses

#### Total Reading Time

There was a reliable effect of Phrase Type and Adjective Condition. Idioms were read significantly more slowly than literal phrases (β = 0.08, SE = 0.02, *t* = 4.26, *p* < 0.001), and phrases containing 1 (β = 0.22, SE = 0.02, *t* = 12.42, *p* < 0.001) and 2 adjectives (β = 0.36, SE = 0.03, *t* = 12.12, *p* < 0.001) were read significantly more slowly than phrases without adjectives. Pairwise comparisons further showed that phrases with 2 adjectives were also read significantly more slowly than phrases with 1 adjective (*p*s < 0.001). Crucially, idioms in ID0, ID1, and ID2 conditions were read significantly more slowly than literal phrases in LIT0, LIT1, and LIT2, respectively (*p*s = 0.003). Moreover, LIT0 phrases were read significantly faster than LIT1 phrases, and LIT1 phrases faster than LIT2 phrases, and equally, ID0 idioms were read significantly faster than ID1 idioms, and ID1 idioms faster than ID2 idioms (*p*s < 0.01).

#### Regression Probability

There was a main effect of Phrase Type and a significant interaction between Phrase Type and Adjective Condition. Idioms had a higher regression probability than literal phrases (β = 0.23, SE = 0.09, *z* = 2.02, *p* = 0.04), and phrases containing 1 (β = 0.39, SE = 0.09, *z* = 4.44, *p* < 0.001) and 2 adjectives (β = 0.58, SE = 0.09, *z* = 6.69, *p* < 0.001) had a higher regression probability than phrases without adjectives. Further pairwise comparisons also showed that phrases with 2 adjectives had a higher regression probability than phrases with 1 adjective (*p*s < 0.001). The interaction in the model showed that idioms in ID2 elicited a significantly higher regression probability than literal phrases in LIT0 (β = 0.31, SE = 0.12, *z* = 2.57, *p* = 0.01). Further pairwise comparisons demonstrated that idioms in ID1, and ID2 had a higher regression probability than literal phrases in LIT1 and LIT2, respectively (*p*s ≤ 0.001), but no differences were noted between ID0 and LIT0 (*p* = 0.64). Further, idioms in ID2 had a higher regression probability than in ID1, and idioms in ID1 had a higher regression probability than in ID0 (*p*s < 0.005). Literal phrases in LIT1 and LIT2 also had a higher regression probability than in LIT0 (*p*s ≥ 0.001), but regression probability between LIT1 and LIT2 conditions did not differ (*p* = 0.33).

### Word-Level Analyses

#### First Pass Gaze Duration

##### Verb region

There was no effect of Phrase Type (β = 0.01, SE = 0.01, *t* = 0.61; *p* = 0.54) or Adjective Condition (β*_1__–__*adjective*_* = 0.00, SE = 0.01, *t* = 0.30, *p* = 0.76; β*_2__–__*adjectives*_* = 0.01, SE = 0.01, *t* = 0.97, *p* = 0.33).

##### Adjective region

There was an effect of Phrase Type: adjectives elicited significantly longer fixations in idioms than in literal phrases (β = 0.02, SE = 0.01, *t* = 1.98, *p* = 0.04), but pairwise comparisons showed that adjectives in ID1 and ID2, did not differ significantly from adjectives in LIT1 and LIT2, respectively (*p*s > 0.28). There was no further effect of Adjective Condition (β*_2__–adjectives_* = −0.01, SE = 0.01, *t* = −0.55, *p* = 0.58).

##### Final word region

There was no reliable effect of Phrase Type (β = 0.01, SE = 0.01, *t* = 1.21, *p* = 0.22), or of the 1-adjective condition (β*_1__–adjective_* = 0.01, SE = 0.01, *t* = 1.21, *p* = 0.22). However, final words in the 2-adjective condition elicited significantly longer fixations than those in the 0-adjective condition in both idioms and literal phrases (β*_2__–adjectives_* = 0.03, SE = 0.01, *t* = 2.86, *p* = 0.004). In addition, no differences were noted in fixation durations between idiom final words in ID0, ID1, and ID2 (*p*s = 1.00).

#### Go-Past Time

##### Verb region

There were no reliable effects of Phrase Type (β = 0.01, SE = 0.02, *t* = 0.40, *p* = 0.68) or Adjective Condition (β*_1__–__*adjective*_* = 0.00, SE = 0.01, *t* = 0.12, *p* = 0.90; β*_2__–__*adjectives*_* = 0.02, SE = 0.01, *t* = 1.36, *p* = 0.17).

##### Adjective region

There was a reliable effect of Phrase Type and Adjective Condition. Adjectives in idioms were read more slowly than adjectives in literal phrases (β = 0.06, SE = 0.01, *t* = 4.33, *p* < 0.001), but adjectives in the 2-adjective condition were read significantly faster than those in the 1-adjective condition in both Phrase Types (β = −0.03, SE = 0.01, *t* = −2.07, *p* = 0.03). It may be that when participants encountered 2 adjectives in sequence, they “skimmed” them quickly without spending as much time attempting to integrate them, at least not until the subsequent context was read. Pairwise comparisons further showed that adjectives in ID1 and ID2 were read significantly more slowly than those in LIT1 and LIT2, respectively (*p*s < 0.002).

##### Final word region

There was a significant effect of Phrase Type: final words in idioms were read significantly more slowly than final words in literal phrases (β = 0.06, SE = 0.01, *t* = 3.98, *p* < 0.001). There was also an effect of Adjective Condition. Final words in the 2-adjective condition were read significantly more slowly than final words in the 0-adjective condition (β = 0.04, SE = 0.01, *t* = 2.79, *p* = 0.005), but there was no reliable difference between final words in the 1- versus the 0-adjective condition (β = 0.02, SE = 0.01, *t* = 1.62, *p* = 0.10). Pairwise comparisons showed that final words in ID0, ID1, and ID2 were read more slowly than final words in LIT0, LIT1, and LIT2, respectively (*p*s < 0.002), but the go-past time of final words in ID01, ID1, and ID2 did not differ significantly from each other (*p*s > 0.9).

#### Total Reading Time

##### Verb region

There was a main effect of Phrase Type. Verbs in idioms were read significantly more slowly than verbs in literal phrases (β = 0.05, SE = 0.01 *t* = 4.30, *p* < 0.001), and an effect of Adjectival Condition: verbs in the 1-adjective (β = 0.03, SE = 0.01, *t* = 2.17, *p* = 0.03) and verbs in the 2-adjective condition (β = 0.05, SE = 0.01, *t* = 3.65, *p* < 0.001) were read significantly faster than those in the 0-adjective condition. Further pairwise comparisons showed no reliable difference between verbs in the 1- versus 2-adjective condition (*p* = 0.43), but verbs in ID0, ID1, and ID2 were read significantly more slowly than verbs in LIT0, LI1, and LIT2, respectively (*p*s < 0.004).

##### Adjective region

There was a main effect of Phrase Type: adjectives in idioms were read more slowly than adjectives in literal phrases (β = 0.05, SE = 0.01, *t* = 4.20, *p* < 0.001). There was no further effect of Adjectival Condition (β = −0.02, SE = 0.01, *t* = −1.74, *p* = 0.08). Pairwise comparisons indicated that adjectives in ID1 and ID2 were read more slowly than adjectives in LIT1 and LIT2, respectively (*p*s < 0.003).

##### Final word

There was a significant effect of Phrase Type whereby final words in idioms were read significantly more slowly than final words in literal phrases (β = 0.03, SE = 0.01, *t* = 2.92, *p* = 0.004). There was also an effect of Adjective Condition, whereby final words in the 2-adjective condition were read more slowly compared to those in the 0-adjective condition (β = 0.03, SE = 0.01, *t* = 2.54, *p* = 0.01), but there was no reliable difference between final words in the 1- versus the 0-adjectives condition (β = 0.02, SE = 0.01, *t* = 1.79, *p* = 0.07). Further pairwise comparisons showed that final words in ID0, ID1, and ID2 were read significantly more slowly than final words in LIT0, LIT1, and LIT2, respectively (*p*s ≤ 0.05), but no significant differences were noted between final words in ID0, ID1, and ID2 (*p*s > 0.9).

#### Predictors

Below we report the effect of the various additional predictors on the phrase- and word-level analyses. Of note, familiarity, literalness, and transparency are only discussed in relation to idioms, as these are fundamentally idiom-specific variables. Frequency and predictability (cloze probability), as well as word/phrase length and trial sequence number, are discussed in relation to both Phrase Types as these variables also apply to literal phrases.

##### Familiarity

There was no reliable effect of familiarity in any models, in any ROI.

##### Literalness

Lower idiom literalness significantly increased the reading time of idiom final words in go-past time (β = 0.02, SE = 0.01, *t* = 2.29, *p* = 0.02), but there were no further effects of literalness in final words or in any other ROIs. The less literal idioms were, the longer total reading time the phrase region tended to elicit, however, the effect was only approaching significance (β = 0.09, SE = 0.05, *t* = 1.80, *p* = 0.07).

##### Transparency

**FIGURE 1:**
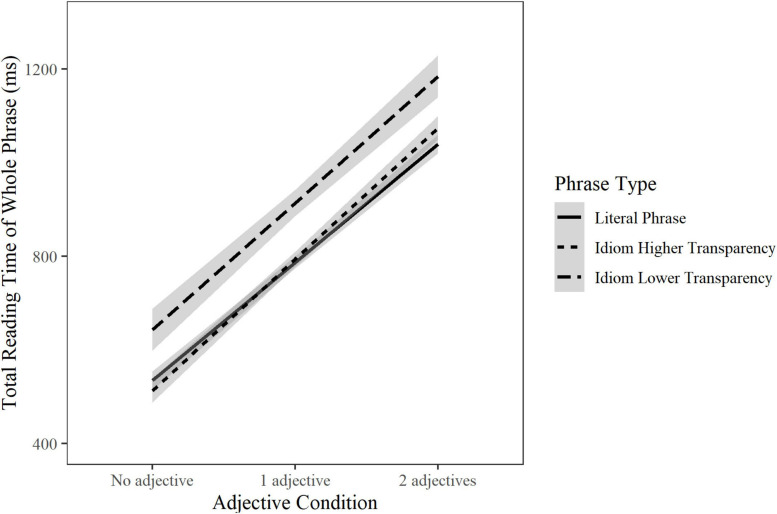
The total reading time in ms of high versus low transparent idioms and matched literal phrases. Highly transparent idioms had a transparency score <3.

Transparency was the only idiom-related factor to consistently affect reading behavior across ROIs. Lower transparency significantly slowed the reading time of idiom verbs in total reading time (β = 0.04, SE = 0.01, *t* = 3.65, *p* < 0.001), of idiom adjectives in all measures (first pass gaze: β = 0.30, SE = 0.01, *t* = 3.89, *p* < 0.001; go-past time: β = 0.04, SE = 0.01, *t* = 3.86, *p* < 0.001; and total reading time: β = 0.06, SE = 0.01, *t* = 6.5, *p* < 0.001), of idiom final words in go-past time (β = 0.03, SE = 0.01, *t* = 3.03, *p* = 0.002) and total reading time (β = 0.03, SE = 0.01, *t* = 3.68, *p* < 0.001), and of whole idiom phrases in total reading time (β = 0.06, SE = 0.01, *t* = 4.38, *p* < 0.001) regardless of Adjective Condition (Figure 1). Lower idiom transparency also increased regression probability into the idiom phrase region (β = 0.21, SE = 0.05, *t* = 4.42, *p* < 0.001).

To better understand the role of transparency relative to the literal phrases, we split idioms into two bins containing high versus low transparency idioms and plotted the total reading time of the phrase region across Adjective Conditions (Figure 1). The trends suggest that more transparent idioms were read significantly faster than less transparent idioms in all Adjective Conditions, but *not* faster than their corresponding literal phrases; instead, transparent idioms were read approximately at the same speed as their equivalent literal phrases.

##### Frequency

**FIGURE 2:**
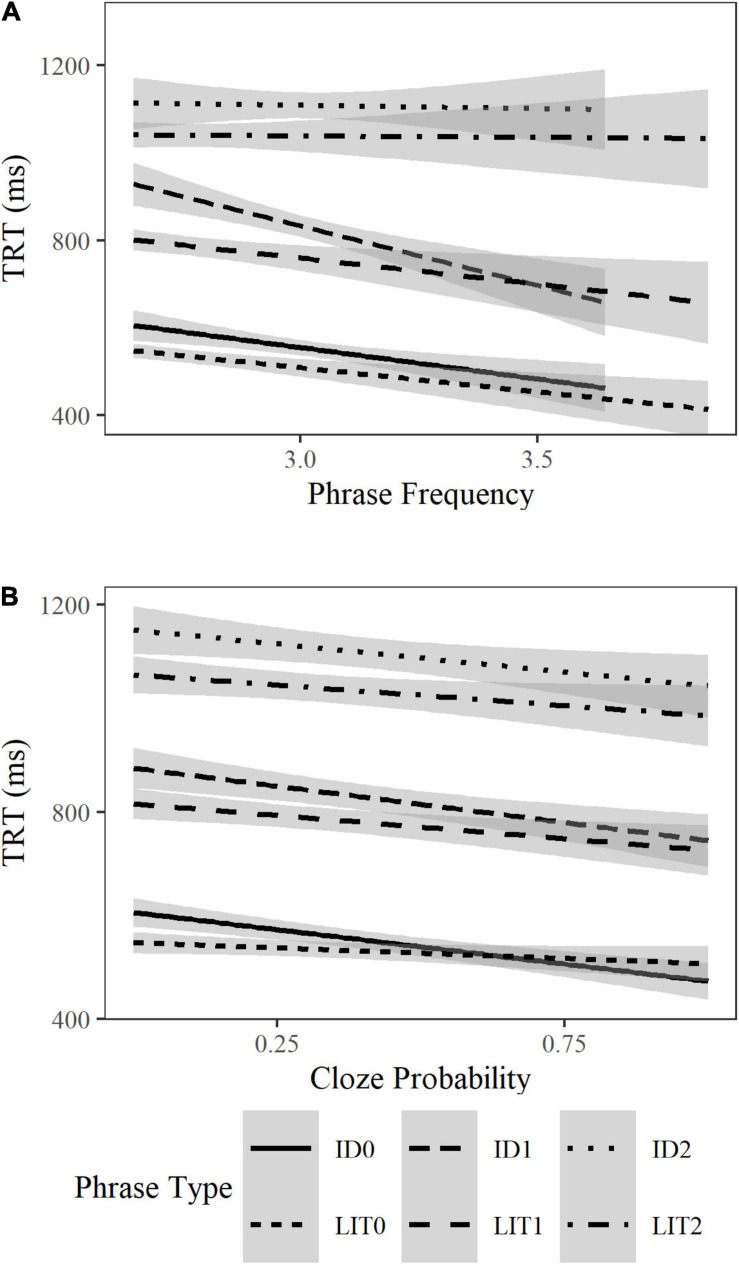
Effects of phrase frequency and cloze probability on the total reading time of idioms and literal phrases (in ms) in the phrase region across all adjective conditions. Phrase frequency values **(A)** are reported on the Zipf scale, and cloze probability **(B)** are reported as a probability between 0 and 1.

Increased verb or phrase frequency, respectively^5^, significantly reduced the reading time of verbs in first pass gaze duration (β = −0.13, SE = 0.04, *t* = −3.17, *p* = 0.002) and total reading time (β = −0.18, SE = 0.05, *t* = −3.55, *p* < 0.001), of adjectives in first pass gaze (β = −0.06, SE = 0.02, *t* = −2.29, *p* = 0.02) and go-past time (β = −0.06, SE = 0.03, *t* = −5.29, *p* = 0.04), and of the whole phrase in total reading time (β = −0.01, SE = 0.03, *t* = −2.78, *p* = 0.005) regardless of Phrase Type. Figure 2A suggests that increased frequency facilitated the processing of idioms and literal phrases in the 0-, or 1-adjective condition, but not those in the 2-adjective condition. Idioms were not read significantly faster than matched literal phrases in any condition regardless of frequency.

##### Cloze probability

Higher cloze probability (of the phrases’ final word) speeded up the reading time of the final word in first pass gaze (β = −0.02, SE = 0.01, *t* = −4.02, *p* < 0.001), go-past time (β = −0.03, SE = 0.01, *t* = −3.42, *p* = 0.001), and total reading time (β = −0.04, SE = 0.01, *t* = −6.31, *p* < 0.001), as well as the total reading time of the phrases regardless of Phrase Type (β = −0.03, SE = 0.01, *t* = −3.76, *p* < 0.001). Higher cloze probability also significantly reduced the probability for a regression into the phrase region regardless of Phrase Type (β = −0.12, SE = 0.03, *t* = −3.42, *p* = 0.001). Figure 2B suggests that increased cloze probability benefited idioms and literal phrases in all adjective conditions. Idioms were not significantly faster to read than matched literal phrases in any condition regardless of cloze probability.

##### Word/phrase length

Increased word length significantly slowed the reading time of verbs in all measures (first pass gaze: β = 0.02, SE = 0.00, *t* = 7.04, *p* < 0.001; go-past time: β = 0.03, SE = 0.01, *t* = 5.0, *p* < 0.001; total reading time: β = 0.02, SE = 0.00, *t* = 5.18, *p* < 0.001), adjectives in all measures (first pass gaze: β = 0.03, SE = 0.00, *t* = 14.673, *p* < 0.001; go-past time: β = 0.02, SE = 0.00, *t* = 7.70, *p* < 0.001; total reading time: β = 0.04, SE = 0.00, *t* = 15.39, *p* < 0.001), and final words in all measures (first pass gaze: β = 0.02, SE = 0.01, *t* = 4.23, *p* < 0.001; go-past time: β = 0.02, SE = 0.01, *t* = 3.59, *p* < 0.001; total reading time: β = 0.02, SE = 0.01, *t* = 4.34, *p* < 0.001), regardless of Phrase Type. Increased phrase length significantly slowed the reading time of phrases in total reading time (β = 0.03, SE = 0.00, *t* = 12.94, *p* < 0.001) regardless of Phrase Type. The effect of word/phrase length on reading speed is well attested in the literature (see Rayner, 2009; Hyönä, 2011; Conklin et al., 2018), and since this predictor was only considered to account for some of the variability in the data, and to check whether the main effects would persist despite it, word/phrase length is not considered in the discussion.

##### Trial sequence number

Higher trial sequence number was associated with faster reading time of verbs in total reading time (β = 0.00, SE = 0.00, *t* = −4.05, *p* < 0.001), of adjectives in all measures (first pass gaze: β = 0.00, SE = 0.00, *t* = −2.41, *p* = 0.01; go-past time: β = 0.00, SE = 0.01, *t* = −5.29, *p* < 0.001; total reading time: β = 0.00, SE = 0.00, *t* = −5.76, *p* < 0.001), of final words in total reading time (β = 0.00, SE = 0.00, *t* = −2.65, *p* = 0.008), and of phrases in total reading time (β = 0.00, SE = 0.00, *t* = −11.30, *p* < 0.001) regardless of Phrase Type. Higher trial sequence number was also associated with a significantly reduced probability for a regression into the phrase region (β = 0.00, SE = 0.00, *z* = −8.95, *p* < 0.001) regardless of Phrase Type. As experience with the stimuli increased, the processing of the items appears to have become easier, but this benefited both Phrase Types and did not impact one condition more than the other. Thus, trial sequence number is not discussed further.

## Discussion

We were interested in whether familiar canonical idioms, and versions of them modified with 1 or 2 adjectives, would exhibit a processing advantage over matched literal phrases when prior context biased the phrases to the appropriate interpretation (figurative or literal, respectively). We compared final word reading times and whole phrase reading times for the idiom and literal conditions. For the idioms, we were interested in whether the final word of modified idioms (1 or 2 adjectives) would exhibit an advantage over the final word of canonical idioms (0 adjectives), as previous research has suggested that adjective insertion reduces the number of possible phrase continuations. To this end we conducted an eye-tracking study to compare the reading of canonical idioms, idioms with 1 and 2 inserted adjectives, and matched literal phrases. Idiom familiarity, literalness, and transparency, as well as phrase frequency and predictability, were examined as predictors to investigate their influence on the processing of canonical and modified idiom forms. We start by summarizing the main findings before moving to a discussion of the potential implications for idiom processing theories and specifically for the idiom processing advantage.

### Modified Idioms Versus Literal Phrases

Our findings suggest that there is no idiom processing advantage when adjectives are inserted into an idiomatic phrase. In the phrase region, idioms modified by 1 and 2 adjectives [*spill the spicy, (red) beans*] elicited significantly longer reading times than literal phrases with 1 and 2 adjectives, respectively [*burn the spicy, (red) beans*]. Further, modified idioms required greater reanalysis relative to their matched controls, being significantly more likely to elicit a regression. Similarly, idiom verbs, adjectives, and final words produced significantly longer total reading times than the equivalents in literal phrases, while idiom adjectives and final words also induced consistently longer fixations in go-past time, indicating greater processing effort already in the early stages of processing. No differences were noted between idiom and literal verbs, adjectives, or final words during first pass gaze duration. In sum, no advantages were noted for modified idioms relative to the literal phrases in any of the regions we examined, in any measure.

Unlike Vilkaitė (2016), who found that collocations with extra words were faster to process than matched non-collocations (*meet/avoid the widely discussed demand*), we did not find a comparable advantage for modified idioms. In Vilkaitė (2016), intervening words consisted mostly of function words with only *some* adjectives and nouns, which could account for the contrasting outcomes. Another important difference is the type of multi-word unit under investigation (i.e., idioms versus collocations). It may be that a formulaic processing advantage can survive modification in other types of multi-word units but not in idioms. The present results lend support to earlier behavioral findings reporting an increase in processing time as a function of adjectival (or other word) insertion (McGlone et al., 1994; Van de Voort and Vonk, 1995), and reinforce Haeuser et al.’s (2020) recent finding, whereby idioms modified by adjectives, and intended in their figurative sense, were read more slowly than matched literal phrases. Importantly, the present results extend this by showing that the processing of adjectivally modified idioms does not improve when a preceding context biases their figurative meaning. This also implies that idiom processing difficulties do not simply originate from the phrases being understood literally [as Haeuser et al. (2020) assumed], as the context pointed to the idiomatic interpretation. This is discussed in more detail below.

### Modified Idioms Versus Canonical Idioms

Idiom final words in the modified conditions [*beans* in *spill the spicy, (red) beans*] did not exhibit an advantage over those in canonical forms (*beans* in *spill the beans*), as they were not faster to read in any measure. It seems, therefore, that the insertion of modifiers did not sufficiently reduce the number of possible phrase continuations as suggested by Molinaro et al. (2013). Thus, the facilitation observed by Molinaro et al. (2013), for nouns following adjectival insertion in modified versus unmodified complex prepositions, was not replicated here with idiom final words. However, our findings do support the claim that the processing of modified forms does not differ significantly from that of canonical versions above and beyond having to read an extra word (Geeraert et al., 2017a). This was evidenced by the fact that fixation durations for idiom verbs and idiom final words in different Adjective Conditions did not differ significantly from one another in any measure, meaning that the longer total reading times observed for modified versus canonical idioms in the phrase region were induced by the extra adjectives alone.

Modified idioms also received significantly more regressions than both unmodified idioms and matched literal phrases, but modified literal phrases also received more regressions than unmodified ones. This suggests that adjectives required reanalysis in both Phrase Types, although adjectives were significantly harder to process in idioms. This is likely due to the need for metaphoric inferencing for the adjectives to be interpreted in idioms, whereas the same adjectives in literal phrases referred to literal/physical properties of the final words, and as such, they required less processing effort.

### Canonical Idioms Versus Literal Phrases

In line with what we observed for modified idioms, we found that canonical idioms (*spill the beans*) were read significantly more slowly than matched literal phrases (*burn the beans*), suggesting that idiom processing came at a cost, with or without adjectives. As in the modified conditions, adjectives and final words in canonical idioms yielded significantly longer fixations in both go-past time and total reading time than in literal phrases, but no differences were noted in first pass gaze duration. Unlike modified idioms, however, canonical idioms were not more (or less) likely to elicit a regression than matched literal phrases, implying that despite their relative slower reading time, canonical idioms did not require more reanalysis than literal phrases. Nevertheless, the slower reading times observed for canonical idioms is clearly in contrast to much of the previous literature (e.g., Ortony et al., 1978; Swinney and Cutler, 1979; Underwood et al., 2004; Conklin and Schmitt, 2008; Tabossi et al., 2009; Vespignani et al., 2010; Siyanova-Chanturia et al., 2011; Rommers et al., 2013; Carrol and Conklin, 2014b, 2017, 2020), and we will return to this point later.

### The Influence of Phrase Characteristics

Higher phrase predictability (here measured as cloze probability of the final word) and frequency facilitated the processing of both idioms and literal phrases in all Adjective Conditions, with one exception: frequency did not influence the processing of either idioms or literal phrases with 2 adjectives. Furthermore, higher transparency facilitated the processing of idioms in all Adjective Conditions. That is, more transparent idioms in the 0-, 1-, and 2-adjective conditions enjoyed faster processing than less transparent idioms in the same condition. This suggests that higher transparency helps, perhaps because the figurative meaning is more “guessable” [or the idiom is more analyzable (Gibbs et al., 1989)], thus potentially also making adjectives less intrusive. There were no reliable effects of familiarity (though our items were generally rated as highly familiar to begin with), and literalness only seemed to affect the go-past reading time of idiom final words, whereby final words of less literal idioms were read more slowly. All these factors were measured in norming tasks for the idioms (and literal phrases where applicable) without adjectives. The fact that measures related to the unmodified idiom did equally well when the phrases were modified with 1 adjective (if not 2), suggests that when expectations are strong (i.e., due to high frequency and predictability), or when the figurative meaning of an idiom is easier to process (i.e., due to higher transparency), expectations remain strong even when idiomatic phrases are modified. These findings are in line with those of Kyriacou et al. (2020), where passive idiom forms benefited from the familiarity, frequency, and predictability of the canonical form. Crucially, the current results further support the idea that the co-occurrence of idiomatic elements might be more important in the processing of idioms than the elements occurring in their canonical order or form.

Importantly, although the idioms in the current study were familiar and mostly transparent, as well as significantly more frequent and predictable than their matched literal phrases, this did not yield the typical idiom processing advantage for either their canonical or the modified forms. At best, we saw that transparent idioms were processed approximately at the same speed as their matched literal phrases with or without adjectives (Figure 2). Clearly, this contrasted with the expectation of an idiom processing advantage and the role of familiarity, literalness, and transparency in it. This will be taken up in the next section.

### Implications for the Idiom Advantage and Idiom Processing Models

Neurolinguistic findings indicate that idioms elicit stronger and more widespread activation in the brain (Lauro et al., 2007; Zempleni et al., 2007; Boulenger et al., 2008, 2012; Mashal et al., 2008). Activating a figurative meaning as well as selecting the “correct” meaning of an ambiguous idiom (e.g., *kick the bucket* meaning “die” or “boot the pail”), should require more processing effort than a compositional unambiguous, literal phrase (*lift the bucket*). The current results support this view, showing that idioms were more difficult to process than matched literal, unambiguous phrases. Importantly, this was not merely a side-effect of idiom modification since canonical idioms (i.e., 0 adjective condition) exhibited the same pattern, nor was it due to our idioms being unfamiliar or infrequent (at least in comparison to the matched controls).

One might argue that the lack of an idiom processing advantage was due to the short length of our idioms and their relatively low predictability, even though idioms were more predictable than their matched control phrases (completion in cloze task: idioms = 43%; literal phrases = 32%). It is assumed that the figurative meaning of short and/or unpredictable idioms becomes activated around 300 ms after the phrase offset (Cacciari and Tabossi, 1988; Fanari et al., 2010). Conversely, predictable idioms (those recognized before the phrase offset) are activated early [Configuration Hypothesis (Cacciari and Tabossi, 1988)], which may lead to faster processing relative to matched literal phrases. Therefore, the slower reading times we observed for our idioms could reflect a delayed idiom activation. To test whether our more predictable items behaved differently, we conducted an exploratory *post hoc* analysis in the phrase region including only idioms that were likely recognized *before* the phrase offset (i.e., idioms whose final words had a cloze probability ≥0.5) and matched literal controls^6^. The analysis showed that these idioms were still significantly slower to read than their matched literal phrases (β = 0.04, SE = 0.02, *t* = 2.40, *p* = 0.01), implying that the findings are not simply due to late idiom recognition/activation. Further, the differences noted in go-past time between idiom and literal phrase components, suggest that idioms were recognized right before or soon after their final word was encountered and that a certain level of semantic retrieval and meaning integration started as soon as the adjective region was reached (or the final word region in unmodified conditions).

An important question is why the current study does not demonstrate the typically reported idiom advantage, as well as how the results can be explained by models of idiom recognition and processing. We believe that the slower reading times we observed reflect the competition between the literal and figurative meaning of the idioms, and specifically the cost associated with meaning selection. Previous idiom studies may have failed to capture such processing costs because they tested idioms in the absence of context in judgment tasks and reaction time studies (Swinney, 1979; Gibbs et al., 1989; Strandburg et al., 1993; Laurent et al., 2006; Tabossi et al., 2009; Carrol and Conklin, 2014b) or when idioms were preceded (and sometimes also followed) by a neutral context (Vespignani et al., 2010; Carrol et al., 2016; Titone et al., 2019; Carrol and Conklin, 2020; Haeuser et al., 2020). In the absence of context, meaning selection is not obligatory since there is no immediate need to select and/or integrate an appropriate interpretation. When a preceding context is neutral, meaning selection and integration may occur outside the idiom region, so that disambiguation occurs in the post-idiom regions that follow, especially if these clarify the intended meaning of the phrase. However, post-idiom regions tend to vary considerably in terms of lexico-syntactic properties (i.e., they are not matched) and are therefore often overlooked in idiom studies because comparisons are difficult if not impossible to make (Titone and Connine, 1999; Titone et al., 2019; Beck and Weber, 2020). Therefore, previous idiom studies may have failed to observe processing difficulties related to idiom processing as they typically focus on the idiom region alone. Furthermore, in studies where both idioms and matched literal phrases were *preceded* by a biasing context, as opposed to a neutral one, the context favored the figurative (and/or literal) meaning of the idioms but made the literal controls semantically anomalous (Conklin and Schmitt, 2008; Rommers et al., 2013; Carrol and Conklin, 2017; Kyriacou et al., 2020), or the literal controls were plausible but did not strictly match idioms in terms of frequency or other lexical properties (Ortony et al., 1978; Underwood et al., 2004; Siyanova-Chanturia et al., 2011). Clearly, if literal phrases are contextually inappropriate, or are poorly matched with respect to idioms, then this will create an idiom advantage. Finally, the location and strength of context has more often been examined in relation to how it might affect the activation of the literal versus the figurative meaning of an idiom (e.g., Titone and Connine, 1994; Fanari et al., 2010; Cacciari and Corradini, 2015; Beck and Weber, 2020), as opposed to the processing of an idiom versus a matched literal phrase.

The current results point to the need for idiom activation and recognition to be distinct from meaning selection (and integration); the former may elicit a processing advantage due to the availability of a lexical route (i.e., direct retrieval of a familiar idiom), while meaning selection and integration might be associated with a processing disadvantage relative to unambiguous literal phrases. Encountering an ambiguous idiom without a prior biasing context may lead to quick recognition/activation of the phrase but meaning selection might be delayed until further information becomes available (in the post-idiom regions). Interestingly, this would mirror findings from the processing of lexically ambiguous words, and specifically of polysemes (e.g., *church* referring to the “building” or the “religions organization”), where meaning selection can be stalled until further information clarifies the intended sense (Frazier and Rayner, 1990; Pickering and Frisson, 2001). Notably, polysemes sometimes exhibit an advantage relative to frequency-matched, unambiguous words (thus resembling the idiom advantage), which is attributed to their sense relatedness (Klepousniotou, 2002; Klepousniotou and Baum, 2007; Klepousniotou et al., 2012). In a similar way, the different meanings of an idiom may be related metaphorically (where transparency/decomposition is high, for instance), and indeed some recent evidence suggests that idioms may behave like polysemes (see Milburn and Warren, 2019). On the other hand, if idioms are preceded by a biasing context which elucidates the intended sense of the phrase (as in the current study), there is no need to postpone meaning selection, and this can occur within the phrase region itself. What is more, if idioms are preceded by disambiguating context *and* are modified, comprehension may be further taxed as the need to select and integrate the correct meaning is further burdened by the need to also process the modification. In other words, the slower processing of both canonical and modified idioms observed in this study may reflect, first, integration happening in the phrase region (as the context guided meaning selection and allowed integration to happen there), and second, that adjective insertion in the modified conditions introduced an additional layer of processing difficulty, leading to yet longer processing times.

In support of such an argument, Colombo (2014) found that when prior context biased the figurative meaning of an idiom, reading times in the phrase region were slower (Experiment 3), whilst when prior context was neutral (Experiment 2) reading times were faster (see also Titone et al., 2019). A recent study by Beck and Weber (2020) did not replicate this finding. However, Beck and Weber’s study, in addition to a preceding biasing context, their idioms were followed by information that either confirmed or disconfirmed original expectations [e.g., “*The new schoolboy who didn’t know anyone in his class just wanted to break the ice* (*with his peers*)/(*on the lake*)” where *on the lake* is anomalous with a figurative interpretation]. This may have led to strategic processing, encouraging participants to keep both meanings activated until the entire sentence had been read. In line with the present findings, an ERP study by Canal et al. (2017) comparing the processing of idioms and literal phrases preceded by biasing context, showed that idioms used figuratively produced an increased positivity effect (PNP), which was not evident for literal phrases or when idioms were used literally. As PNP effects are associated with sentence revision mechanisms, the researchers concluded that in the case of idioms intended figuratively, the need to select the appropriate meaning and adjust it to the sentence representation requires “enriched integration.” Moreover, the researchers failed to observe the usual N400 reduction for idiom elements, just as the current study failed to demonstrate an idiom advantage in terms of faster reading times, reinforcing the idea that an advantage may only manifest in idiom recognition and activation, and/or when the phrases are preceded by neutral (or no) context.

In the current study, we have demonstrated that adding adjectives increases processing times for idioms (but also for literal phrases), reflecting the need to process the additional information. Although modified idioms in the 1- and 2-adjective conditions also received more regressions relative to their matched literal phrases – thus suggesting more problematic adjective integration – fixations to idiom verbs and final words did not differ in different Adjective Conditions. This implies that modified idioms were recognized as idioms and that their figurative meaning was activated despite the addition of adjectives. Thus, our findings are most in line with hybrid models of idiom representation (Cacciari and Tabossi, 1988; Sprenger et al., 2006; Libben and Titone, 2008), since these consider the impact of both the component words and the idiom configuration itself and can therefore accommodate idiom modification. However, these models were designed to explain how an idiom becomes activated and do not make specific predictions about how or when an idiom’s meaning might be integrated as part of wider discourse (with or without modifications). Therefore, these models cannot fully account for the present data. Notably, in this study we assumed the widely attested idiom advantage would be present and aimed to test whether it would survive modification. The lack of a clear idiom advantage demonstrates the need for further research, in particular exploring the role of context, as well as the nature of the matched, compositional control phrase, when drawing conclusions about an idiom advantage. Future studies will need to continue to explore the role of context and modification and models will need to be adapted to accommodate such findings.

## Conclusion

The present results are best explained by differentiating idiom activation from meaning selection and integration. Our idioms were familiar, used in their figurative sense, and were preceded by context that foregrounded this meaning. The literal control phrases were equally felicitous in their respective contexts. Contrary to our hypotheses, and the wider literature, the observed reading times were slower for idioms with and without adjectives compared to matched literal phrases. The current results demonstrate the role of the “matched control phrase” and the biasing context in underpinning the idiom advantage. When a preceding context biases an idiomatic meaning or that of a literal phrase, there is no idiom advantage. In fact, processing of idioms is slower, likely because of the competition between an idiom’s literal and figurative senses. Conversely, a literal, unambiguous phrase only has one plausible interpretation and hence integrating this meaning is less effortful. In other studies, where the biasing context appears later in the sentence (after the idiom) or is not present, meaning integration may occur outside the idiom region. Thus, any processing difficulties may “fall off the radar” as the phrase region is often the sole focus of analyses. Furthermore, while factors such as high familiarity and predictability may contribute to idiom recognition, and hence fast activation, they do not make meaning integration easier for idioms relative to literal, unambiguous phrases. Finally, while adjectives in idioms are more difficult to interpret, as they involve complex inferencing, adjective insertion in idioms does not seem to inhibit recognition and activation of the phrase, nor does it result in loss of the figurative interpretation of the idiom.

## Data Availability Statement

The raw data supporting the conclusions of this article will be made available by the authors, without undue reservation.

## Ethics Statement

The studies involving human participants were reviewed and approved by the Faculty of Arts Ethics, University of Nottingham. The patients/participants provided their written informed consent to participate in this study.

## Author Contributions

MK and KC contributed to the conception and design of the study. MK, KC, and DT contributed to the stimuli design. MK designed the study, collected and analyzed the data, and wrote the first full draft of the manuscript. KC contributed to substantial revisions of the original draft. All authors read, reviewed, and approved the submitted version.

## Conflict of Interest

The authors declare that the research was conducted in the absence of any commercial or financial relationships that could be construed as a potential conflict of interest.

## Publisher’s Note

All claims expressed in this article are solely those of the authors and do not necessarily represent those of their affiliated organizations, or those of the publisher, the editors and the reviewers. Any product that may be evaluated in this article, or claim that may be made by its manufacturer, is not guaranteed or endorsed by the publisher.

## Footnotes

1 Of note, while passive idioms in Kyriacou et al. (2020) demonstrated a processing advantage relative to matched literal phrases, the latter were always anomalous in the context (e.g., seeing *the apple was kicked* where only *the bucket was kicked* would make sense). Thus, the idiom advantage could have been conflated with contextual plausibility and does not entail that passive literal phrases are slower to process when they are contextually plausible.

2 Frequency values reported throughout the paper were taken from the BNC (British National Corpus, 2007) and transformed using the Zipf scale (see van Heuven et al., 2014).

3 Here, skipping refers to instances where the ROI was skipped during first pass reading and was not later visited by a regressive fixation. Skipping rates were largely balanced between Phrase Type (and Adjective Condition): verbs: 9.1% in idioms versus 7.4% in literal phrases; adjectives: 8.9% in idioms versus 9.9% in literal phrases; final words: 11.8% in idioms versus 13.2% in literal phrases.

4 For idiom final words we also examined skipping, as these words are sometimes skipped during first pass reading time indicating easier processing (Carrol and Conklin, 2017). Here, idiom final words were not more likely to be skipped than literal final words, nor were final words of modified idioms more likely to be skipped than those in canonical forms. Therefore, we do not report the results of the skipping analysis.

5 Verb frequency was included in verb region models as verbs differed between idiom and literal phrases, while phrase frequency was included in all the models for the remaining ROIs.

6 These idioms were still significantly more predictable and frequent than their matched literal phrases, as well as highly familiar, non-literal, and transparent.
