# Supplementary material for: When the Idiom Advantage Comes Up Short: Eye-Tracking Canonical and Modified Idioms
Source: Front Psychol. 2021 Aug 2;12:675046. doi: 10.3389/fpsyg.2021.675046 (PMC8364978; doi:10.3389/fpsyg.2021.675046)
Supplement: Supplementary file 1 [file Data_Sheet_1.docx]

**Supplementary material**

Outputs of final converging models for the Phrase, Verb, Adjective, and Final Word regions.

| **Phrase Region** | | | | | | | | | | | |  |  |  |
| --- | --- | --- | --- | --- | --- | --- | --- | --- | --- | --- | --- | --- | --- | --- |
|  | **Total Reading Time** | | | | |  | **Regression Probability** | | | | |  |  |  |
| *Predictors* | *b* | *SE* | *Ci* | *t* | *p* |  | *b* | *SE* | *Ci* | *z* | *p* |  |  |  |
| (Intercept) | 6.06 | 0.11 | 5.85 – 6.27 | 56.71 | **<0.001** |  | -0.75 | 0.11 | -0.95 – -0.54 | -7.03 | **<0.001** |  |  |  |
| Phrase Type [Idiom] | 0.08 | 0.02 | 0.04 – 0.12 | 4.26 | **<0.001** |  | 0.2 | 0.1 | 0.01 – 0.39 | 2.02 | **0.043** |  |  |  |
| Adjective Condition [1 Adjective] | 0.22 | 0.02 | 0.19 – 0.26 | 12.42 | **<0.001** |  | 0.39 | 0.09 | 0.22 – 0.56 | 4.46 | **<0.001** |  |  |  |
| Adjective Condition [2 Adjectives] | 0.36 | 0.03 | 0.30 – 0.42 | 12.12 | **<0.001** |  | 0.58 | 0.09 | 0.41 – 0.75 | 6.69 | **<0.001** |  |  |  |
| Phrase Length | 0.03 | 0 | 0.02 – 0.03 | 12.94 | **<0.001** |  |  |  |  |  |  |  |  |  |
| Cloze Probability | -0.03 | 0.01 | -0.05 – -0.02 | -3.76 | **<0.001** |  | -0.12 | 0.03 | -0.19 – -0.05 | -3.42 | **0.001** |  |  |  |
| Frequency | -0.1 | 0.03 | -0.17 – -0.03 | -2.78 | **0.005** |  |  |  |  |  |  |  |  |  |
| Trial Sequence Number | 0 | 0 | -0.00 – -0.00 | -11.3 | **<0.001** |  | 0 | 0 | -0.01 – -0.00 | -8.9 | **<0.001** |  |  |  |
| Literalness | 0.02 | 0.01 | -0.00 – 0.05 | 1.6 | 0.111 |  | 0.09 | 0.05 | -0.01 – 0.18 | 1.8 | 0.071 |  |  |  |
| Transparency | 0.06 | 0.01 | 0.03 – 0.08 | 4.38 | **<0.001** |  | 0.21 | 0.05 | 0.12 – 0.31 | 4.42 | **<0.001** |  |  |  |
| Phrase Type [Idiom] * Adjective Condition [1 Adjective] |  |  |  |  |  |  | 0.16 | 0.12 | -0.08 – 0.40 | 1.29 | 0.196 |  |  |  |
| Phrase Type [Idiom] * Adjective Condition [2 Adjectives] |  |  |  |  |  |  | 0.31 | 0.12 | 0.07 – 0.55 | 2.57 | **0.01** |  |  |  |
| **Random Effects** | | | | | | | | | | | |  |  |  |
| Σ^2^ | 0.15 | | | | |  | 3.29 | | | | |  |  |  |
| Τ_00_ | 0.06 _PPT_ | | | | |  | 0.38 _PPT_ | | | | |  |  |  |
|  | 0.01 _ITEM_ | | | | |  | 0.10 _ITEM_ | | | | |  |  |  |
| Τ_11_ | 0.00 _PPT.IDIOMATICITYIDIOM_ | | | | |  | 0.00 _PPT.IDIOMATICITYIDIOM_ | | | | |  |  |  |
|  | 0.02 _ITEM.IDIOMATICITYIDIOM_ | | | | |  | 0.12 _ITEM.IDIOMATICITYIDIOM_ | | | | |  |  |  |
| Ρ_01_ | 0.62 _PPT_ | | | | |  | 0.96 _PPT_ | | | | |  |  |  |
|  | -0.65 _ITEM_ | | | | |  | -0.69 _ITEM_ | | | | |  |  |  |
| Icc | 0.34 | | | | |  | 0.13 | | | | |  |  |  |
| N | 90 _PPT_ | | | | |  | 90 _PPT_ | | | | |  |  |  |
|  | 90 _ITEM_ | | | | |  | 90 _ITEM_ | | | | |  |  |  |
| Observations | 8084 | | | | |  | 8084 | | | | |  |  |  |
|  |  |  |  |  |  |  |  |  |  |  |  |  |  |  |
|  |  |  |  |  |  |  |  |  |  |  |  |  |  |  |
|  |  |  |  |  |  |  |  |  |  |  |  |  |  |  |
| **Verbs** | | | | | | | | | | | | | | |
|  | **First Pass Gaze Duration** | | | |  | **Go-Past Reading Time** | | | |  | **Total Reading Time** | | | |
| *Predictors* | *b* | *SE* | *t* | *p* |  | *b* | *SE* | *t* | *p* |  | *b* | *SE* | *t* | *p* |
| (Intercept) | 5.65 | 0.13 | 44.01 | **<0.001** |  | 5.4 | 0.04 | 133.5 | **<0.001** |  | 5.97 | 0.17 | 36.13 | **<0.001** |
| Phrase Type [Idiom] | 0.01 | 0.01 | 0.61 | 0.54 |  | 0.01 | 0.02 | 0.4 | 0.689 |  | 0.05 | 0.01 | 4.3 | **<0.001** |
| Adjective Condition [1 Adjective] | 0 | 0.01 | 0.3 | 0.766 |  | 0 | 0.01 | 0.12 | 0.904 |  | 0.03 | 0.01 | 2.17 | **0.03** |
| Adjective Condition [2 Adjectives] | 0.01 | 0.01 | 0.97 | 0.331 |  | 0.02 | 0.01 | 1.36 | 0.174 |  | 0.05 | 0.01 | 3.65 | **<0.001** |
| Word Length | 0.02 | 0 | 7.04 | **<0.001** |  | 0.03 | 0.01 | 5 | **<0.001** |  | 0.02 | 0 | 5.18 | **<0.001** |
| Frequency | -0.13 | 0.04 | -3.17 | **0.002** |  |  |  |  |  |  | -0.18 | 0.05 | -3.55 | **<0.001** |
| Trial Sequence Number |  |  |  |  |  |  |  |  |  |  | 0 | 0 | -4.05 | **<0.001** |
| Literalness |  |  |  |  |  |  |  |  |  |  | 0.01 | 0.01 | 1.32 | 0.187 |
| Transparency |  |  |  |  |  |  |  |  |  |  | 0.04 | 0.01 | 3.65 | **<0.001** |
| **Random Effects** | | | | | | | | | | | | | | |
| Σ^2^ | 0.13 | | | |  | 0.22 | | | |  | 0.2 | | | |
| Τ_00_ | 0.02 _PPT_ | | | |  | 0.03 _PPT_ | | | |  | 0.03 _PPT_ | | | |
|  | 0.00 _ITEM_ | | | |  | 0.01 _ITEM_ | | | |  | 0.01 _ITEM_ | | | |
| Τ_11_ |  | | | |  | 0.00 _PPT.IDIOMATICITYIDIOM_ | | | |  |  | | | |
|  |  | | | |  | 0.01 _ITEM.IDIOMATICITYIDIOM_ | | | |  |  | | | |
| Ρ_01_ |  | | | |  | -0.10 _PPT_ | | | |  |  | | | |
|  |  | | | |  | -0.38 _ITEM_ | | | |  |  | | | |
| Icc | 0.13 | | | |  | 0.15 | | | |  | 0.16 | | | |
| N | 90 _PPT_ | | | |  | 90 _PPT_ | | | |  | 90 _PPT_ | | | |
|  | 90 _ITEM_ | | | |  | 90 _ITEM_ | | | |  | 90 _ITEM_ | | | |
| Observations | 6738 | | | |  | 6743 | | | |  | 6747 | | | |
|  |  |  |  |  |  |  |  |  |  |  |  |  |  |  |
|  |  |  |  |  |  |  |  |  |  |  |  |  |  |  |
|  |  |  |  |  |  |  |  |  |  |  |  |  |  |  |
| **Adjectives** | | | | | | | | | | | | | | |
|  | **First Pass Gaze Duration** | | | |  | **Go-Past Reading Time** | | | |  | **Total Reading Time** | | | |
| *Predictors* | *b* | *SE* | *t* | *p* |  | *b* | *SE* | *t* | *p* |  | *b* | *SE* | *t* | *p* |
| (Intercept) | 5.43 | 0.07 | 75.3 | **<0.001** |  | 5.64 | 0.09 | 64.77 | **<0.001** |  | 5.5 | 0.08 | 67.57 | **<0.001** |
| Phrase Type [Idiom] | 0.02 | 0.01 | 1.98 | **0.048** |  | 0.06 | 0.01 | 4.33 | **<0.001** |  | 0.05 | 0.01 | 4.2 | **<0.001** |
| Adjective Condition [2 Adjectives] | -0.01 | 0.01 | -0.55 | 0.58 |  | -0.03 | 0.01 | -2.07 | **0.039** |  | -0.02 | 0.01 | -1.74 | 0.082 |
| Word Length | 0.03 | 0 | 14.73 | **<0.001** |  | 0.02 | 0 | 7.7 | **<0.001** |  | 0.04 | 0 | 15.39 | **<0.001** |
| Frequency | -0.06 | 0.02 | -2.29 | **0.022** |  | -0.06 | 0.03 | -2.05 | **0.041** |  | -0.05 | 0.03 | -1.93 | 0.053 |
| Trial Sequence Number | 0 | 0 | -2.41 | **0.016** |  | 0 | 0 | -5.29 | **<0.001** |  | 0 | 0 | -5.76 | **<0.001** |
| Transparency | 0.03 | 0.01 | 3.89 | **<0.001** |  | 0.04 | 0.01 | 3.86 | **<0.001** |  | 0.06 | 0.01 | 6.5 | **<0.001** |
| Literalness |  |  |  |  |  | 0.02 | 0.01 | 1.85 | 0.065 |  | 0 | 0.01 | 0.32 | 0.746 |
| **Random Effects** | | | | | | | | | | | | | | |
| Σ^2^ | 0.13 | | | |  | 0.22 | | | |  | 0.18 | | | |
| Τ_00_ | 0.02 _PPT_ | | | |  | 0.02 _PPT_ | | | |  | 0.03 _PPT_ | | | |
|  | 0.00 _ITEM_ | | | |  | 0.00 _ITEM_ | | | |  | 0.01 _ITEM_ | | | |
| Τ_11_ | 0.00 _PPT.IDIOMATICITYIDIOM_ | | | |  |  | | | |  |  | | | |
|  | 0.00 _ITEM.IDIOMATICITYIDIOM_ | | | |  |  | | | |  |  | | | |
| Ρ_01_ | 0.38 _PPT_ | | | |  |  | | | |  |  | | | |
|  | -0.54 _ITEM_ | | | |  |  | | | |  |  | | | |
| Icc | 0.14 | | | |  | 0.1 | | | |  | 0.16 | | | |
| N | 90 _PPT_ | | | |  | 90 _PPT_ | | | |  | 90 _PPT_ | | | |
|  | 90 _ITEM_ | | | |  | 90 _ITEM_ | | | |  | 90 _ITEM_ | | | |
| Observations | 6573 | | | |  | 6570 | | | |  | 6574 | | | |
|  |  |  |  |  |  |  |  |  |  |  |  |  |  |  |
|  |  |  |  |  |  |  |  |  |  |  |  |  |  |  |
|  |  |  |  |  |  |  |  |  |  |  |  |  |  |  |
| **Final Word** | | | | | | | | | | | | | | |
|  | **First Pass Gaze Duration** | | | |  | **Go-Past Reading Time** | | | |  | **Total Reading Time** | | | |
| *Predictors* | *b* | *SE* | *t* | *p* |  | *b* | *SE* | *t* | *p* |  | *b* | *SE* | *t* | *p* |
| (Intercept) | 5.26 | 0.03 | 169.27 | **<0.001** |  | 5.47 | 0.09 | 58.63 | **<0.001** |  | 5.33 | 0.04 | 148.07 | **<0.001** |
| Phrase Type [Idiom] | 0.01 | 0.01 | 1.21 | 0.228 |  | 0.06 | 0.01 | 3.98 | **<0.001** |  | 0.03 | 0.01 | 2.92 | **0.004** |
| Adjective Condition [1 Adjective] | 0.01 | 0.01 | 1.21 | 0.228 |  | 0.02 | 0.01 | 1.62 | 0.105 |  | 0.02 | 0.01 | 1.79 | 0.074 |
| Adjective Condition [2 Adjectives] | 0.03 | 0.01 | 2.86 | **0.004** |  | 0.04 | 0.01 | 2.79 | **0.005** |  | 0.03 | 0.01 | 2.54 | **0.011** |
| Word Length | 0.02 | 0.01 | 4.23 | **<0.001** |  | 0.02 | 0.01 | 3.59 | **<0.001** |  | 0.02 | 0.01 | 4.34 | **<0.001** |
| Cloze Probability | -0.02 | 0.01 | -4.02 | **<0.001** |  | -0.03 | 0.01 | -3.42 | **0.001** |  | -0.04 | 0.01 | -6.31 | **<0.001** |
| Literalness | 0.02 | 0.01 | 1.94 | 0.052 |  | 0.02 | 0.01 | 2.29 | **0.022** |  |  |  |  |  |
| Transparency | 0.01 | 0.01 | 1.35 | 0.176 |  | 0.03 | 0.01 | 3.03 | **0.002** |  | 0.03 | 0.01 | 3.68 | **<0.001** |
| Frequency |  |  |  |  |  | -0.05 | 0.03 | -1.51 | 0.132 |  |  |  |  |  |
| Trial Sequence Number |  |  |  |  |  |  |  |  |  |  | 0 | 0 | -2.65 | **0.008** |
| **Random Effects** | | | | | | | | | | | | | | |
| Σ^2^ | 0.12 | | | |  | 0.21 | | | |  | 0.16 | | | |
| Τ_00_ | 0.01 _PPT_ | | | |  | 0.02 _PPT_ | | | |  | 0.02 _PPT_ | | | |
|  | 0.00 _ITEM_ | | | |  | 0.00 _ITEM_ | | | |  | 0.00 _ITEM_ | | | |
| Icc | 0.12 | | | |  | 0.1 | | | |  | 0.13 | | | |
| N | 90 _PPT_ | | | |  | 90 _PPT_ | | | |  | 90 _PPT_ | | | |
|  | 90 _ITEM_ | | | |  | 90 _ITEM_ | | | |  | 90 _ITEM_ | | | |
| Observations | 6058 | | | |  | 6057 | | | |  | 6058 | | | |
