# Supplementary material for: When the Idiom Advantage Comes Up Short: Eye-Tracking Canonical and Modified Idioms
Source: Front Psychol. 2021 Aug 2;12:675046. doi: 10.3389/fpsyg.2021.675046 (PMC8364978; doi:10.3389/fpsyg.2021.675046)
Supplement: Supplementary file 2 [file Data_Sheet_2.docx]

**Supplementary Appendix**

List of Idioms, Literal Phrases, and Relevant Adjectives Used in the Experiment

| **No.** | **Idiom** | **Literal Phrase** | **Adjectives** |
| --- | --- | --- | --- |
| **1** | Hit the bottle | Fill the bottle | Small, clear |
| **2** | Grease the wheels | Change the wheels | Sad, wretched |
| **3** | Count the cost | Calculate the cost | High, overall |
| **4** | Draw the line | Erase the line | Fine, narrow |
| **5** | Set the pace | Change the pace | Fast, competitive |
| **6** | Scratch the surface | Polish the surface | Thin, outer |
| **7** | Spill the beans | Burn the beans | Spicy, red |
| **8** | Tighten your belt | Wear your belt | Firm, dependable |
| **9** | Pick a fight | Prevent a fight | Big, silly |
| **10** | Twist one’s arm | Cut one’s arm | Strong, muscled |
| **11** | Keep your head | Soothe your head | Poor, little |
| **12** | Hit the road | Block the road | Long, slow |
| **13** | Beat your chest | Shave your chest | Large, broad |
| **14** | Drag your feet | Stomp your feet | Small, little |
| **15** | Earn your spurs | Break your spurs | Big, shiny |
| **16** | Fan the flames | Douse the flames | Big, red |
| **17** | Chew the fat | Smell the fat | Thick, juicy |
| **18** | Stay the course | Attend the course | Short, intensive |
| **19** | Button your lip | Bite your lip | Thin, red |
| **20** | Line your pockets | Fill your pockets | Large, deep |
| **21** | Tip the balance | Address the balance | Delicate, unstable |
| **22** | Drop the ball | Miss the ball | Easy, final |
| **23** | Bury the hatchet | Sell the hatchet | Old, rusty |
| **24** | Pick one’s brain | Examine one’s brain | Entire, unfortunate |
| **25** | Turn the tables | Place the tables | Trivial, maddening |
| **26** | Kick the bucket | Inhabit the bucket | Horrible, old |
| **27** | Rule the roost | Clean the roost | Big, old |
| **28** | Drop a bombshell | Find a bombshell | Big, powerful |
| **29** | Bite the dust | Blow the dust | Thick, irritating |
| **30** | Hit the wall | Crack the wall | Solid, brick |
| **31** | Move the goalposts | Sell the goalposts | Useless, old |
| **32** | Miss the boat | Sell the boat | Big, costly |
| **33** | Play the game | Cancel the game | Long, tough |
| **34** | Break the mould | Buy the mould | Old, traditional |
| **35** | Learn the ropes | Use the ropes | Long, safety |
| **36** | Pull your weight | Lift your weight | Full, complete |
| **37** | Fit the bill | Pay the bill | Whole, considerable |
| **38** | Bite the bullet | Extract the bullet | Big, awful |
| **39** | Pop the question | Answer the question | Big, important |
| **40** | Waste your breath | Hold your breath | Ragged, valuable |
| **41** | Know the ropes | Unpack the ropes | Twisted, old |
| **42** | Know the score | Keep the score | Full, running |
| **43** | Strike a chord | Play a chord | Sudden, dissonant |
| **44** | Deliver the goods | Order the goods | Main, essential |
| **45** | Eat your words | Change your words | Unfitting, inaccurate |
| **46** | Prepare the ground | Clear the ground | Rough, difficult |
| **47** | Cook the books | Dust the books | Old, cryptic |
| **48** | Make the grade | Erase the grade | Final, high |
| **49** | Flex your muscles | Strain your muscles | Big, strong |
| **50** | Bend the rules | Follow the rules | Strict, legal |
| **51** | Crack the whip | Steal the whip | Loud, painful |
| **52** | Find your feet | Rub your feet | Old, steady |
| **53** | Break the ice | Crush the ice | Substantial, hard |
| **54** | Smell a rat | Find a rat | Big, ugly |
| **55** | Blow a fuse | Buy a fuse | Small, vital |
| **56** | Hit the deck | Clean the deck | Tough, hard |
| **57** | Weather the storm | Avoid the storm | Tough, bitter |
| **58** | Square the circle | Break the circle | Big, awkward |
| **59** | Twiddle one’s thumbs | Squeeze one’s thumbs | Tiny, little |
| **60** | Take a bath | Have a bath | Horrible, unpleasant |
| **61** | Stem the tide | Examine the tide | Strong, rising |
| **62** | Cook your goose | Lose your goose | Big, fat |
| **63** | Twist the knife | Sharpen the knife | Long, dull |
| **64** | Touch a nerve | Hit a nerve | Large, sensitive |
| **65** | Make a splash | Feel a splash | Big, sudden |
| **66** | Lick your wounds | Treat your wounds | Deep, gaping |
| **67** | Lose your marbles | Find your marbles | Bright, precious |
| **68** | Hold the fort | Guard the fort | Small, vulnerable |
| **69** | Steal the show | Sabotage the show | Big, splendid |
| **70** | Face the music | Endure the music | Loud, harsh |
| **71** | Sugar the pill | Take the pill | Large, bitter |
| **72** | Clear the air | Freshen the air | Thick, unpleasant |
| **73** | Carry the day | Spend the day | Long, stressful |
| **74** | Tie the knot | Cut the knot | Huge, tight |
| **75** | Hedge your bets | Double your bets | Big, heavy |
| **76** | Cut your teeth | Brush your teeth | Small, baby |
| **77** | Turn the page | Tear the page | Whole, troubling |
| **78** | Pull the strings | Detangle the strings | Numerous, complex |
| **79** | Rock the boat | Sell the boat | Little, old |
| **80** | Pull one’s leg | Lick one’s leg | Long, tolerant |
| **81** | Jump the gun | Fire the gun | Big, dangerous |
| **82** | Break the bank | Rob the bank | Small, unfortunate |
| **83** | Hit the roof | Damage the roof | High, lofty |
| **84** | Lose the plot | Forget the plot | Basic, original |
| **85** | Have a ball | Hold a ball | Enormous, entertaining |
| **86** | Go the distance | Travel the distance | Long, total |
| **87** | Get the picture | Falsify the picture | Entire, grim |
| **88** | Miss the mark | Draw the mark | Big, wide |
| **89** | Turn the corner | Clear the corner | Dark, tight |
| **90** | Cross the line | Ruin the line | Thin, narrow |
